# Genome-Wide Identification and Analysis of Drought-Responsive Genes and MicroRNAs in Tobacco

**DOI:** 10.3390/ijms16035714

**Published:** 2013-03-12

**Authors:** Fuqiang Yin, Cheng Qin, Jian Gao, Ming Liu, Xirong Luo, Wenyou Zhang, Hongjun Liu, Xinhui Liao, Yaou Shen, Likai Mao, Zhiming Zhang, Haijian Lin, Thomas Lübberstedt, Guangtang Pan

**Affiliations:** 1 School of Agricultural Sciences, Xichang College, Xichang 615000, China; E-Mails: yinfuqiang04@sina.com (F.Y.); lming311608@sina.com (M.L.); zhangwy@gmail.com (W.Z.); 2 Maize Research Institute of Sichuan Agricultural University/Key Laboratory of Biology and Genetic Improvement of Maize in Southwest Region, Ministry of Agriculture, Chengdu 611130, China; E-Mails: cheng.qin.sicau@gmail.com (C.Q.); gaojian8888@gmail.com (J.G.); lhj20305@gmail.com (H.L.); shenyaou@gmail.com (Y.S.); zzmmaize@gmail.com (Z.Z.); linhj521@gmail.com (H.L.); 3 Zunyi Academy of Agricultural Sciences, Zunyi 563102, China; E-Mail: luoxirong1982@gmail.com; 4 Beijing Genomics Institute-Shenzhen, Shenzhen 518083, China; E-Mails: liaoxinhui@bgitechsolutions.com (X.L.); maolikai@bgitechsolutions.com (L.M.); 5 Department of Agronomy, Iowa State University, Ames, IA 50011, USA; E-Mail: thomasl@iastate.edu

**Keywords:** *Nicotiana benthamiana*, DGE, small RNA sequencing, drought responsive genes (DRGs), TFs, gene regulatory network

## Abstract

Drought stress response is a complex trait regulated at transcriptional and post-transcriptional levels in tobacco. Since the 1990s, many studies have shown that miRNAs act in many ways to regulate target expression in plant growth, development and stress response. The recent draft genome sequence of *Nicotiana benthamiana* has provided a framework for Digital Gene Expression (DGE) and small RNA sequencing to understand patterns of transcription in the context of plant response to environmental stress. We sequenced and analyzed three Digital Gene Expression (DGE) libraries from roots of normal and drought-stressed tobacco plants, and four small RNA populations from roots, stems and leaves of control or drought-treated tobacco plants, respectively. We identified 276 candidate drought responsive genes (DRGs) with sequence similarities to 64 known DRGs from other model plant crops, 82 were transcription factors (TFs) including WRKY, NAC, ERF and bZIP families. Of these tobacco DRGs, 54 differentially expressed DRGs included 21 TFs, which belonged to 4 TF families such as NAC (6), MYB (4), ERF (10), and bZIP (1). Additionally, we confirmed expression of 39 known miRNA families (122 members) and five conserved miRNA families, which showed differential regulation under drought stress. Targets of miRNAs were further surveyed based on a recently published study, of which ten targets were DRGs. An integrated gene regulatory network is proposed for the molecular mechanisms of tobacco root response to drought stress using differentially expressed DRGs, the changed expression profiles of miRNAs and their target transcripts. This network analysis serves as a reference for future studies on tobacco response stresses such as drought, cold and heavy metals.

## 1. Introduction

Tobacco, *Nicotiana benthamiana*, is an important agricultural and economic crop in China [1]. It is one of the most commonly used species to study molecular plant-microbe interactions [2,3]. Numerous efforts are underway to improve plant productivity, including reduction of yield loss to environmental stress, such as drought, water logging, salt loading, and freezing. These stresses have adverse effects on plant growth, and plant stress responses are regulated by multiple signaling pathways [4,5]. In general, plants respond and adapt to stress through various biochemical and physiological processes, thereby acquiring stress tolerance [6].

Decades of research into the effects of drought on plant physiology and development have generated a plethora of information. It has been shown that regulatory networks of gene expression under drought and cold stress are active [6], and that particular transcription factors are employed to enhance drought tolerance in plants [7]. Drought stress increases endogenous abscisic acid (ABA) levels and induces ABA-dependent and ABA-independent transcriptional regulatory networks [8]. Many of these responses can be mimicked by external application of ABA or polyethylene glycol (PEG) [6,9]. Drought-responsive transcriptional networks have been primarily developed from related studies in Arabidopsis, and some important promoter elements were confirmed experimentally, such as ABA-responsive elements (ABREs) and coupling elements (CE) [10,11,12]. However, the networks that underlie these responses in tobacco have not been fully characterized.

In addition to drought responsive genes (DRGs) and those transcription factors mentioned, microRNAs (miRNAs) are short (20–22 nt), endogenously expressed, non-translated RNAs that function in posttranscriptional gene regulation. The mature miRNA form is processed from a larger primary transcript (pri-miRNA) [13] and incorporated into the RNA-induced silencing complex (RISC). RISC directs binding to complementary mRNA transcripts, followed by target cleavage or reversible inhibition of translation [14]. MiRNAs act in a variety of ways and they can spatially restrict, temporally regulate, dampen or mutually exclude the expression of their target gene [14]. MiRNAs play critical roles in different developmental pathways, including root [15,16] and shoot development [17]. Additionally, they respond to plant hormones [18], and a variety of stresses like drought, salt, cold and oxidative stress [19,20,21,22]. For instance, ABA treatment and drought stress induces miR159 to accumulate and target MYB TFs that positively regulate ABA responses during seed germination in Arabidopsis, with miR159 as a negative feedback regulator of ABA responses [23]. Zhao *et al.* [21] reported that miRNA169g accumulates during drought stress and miR169g is regulated by dehydration responsive element (DREs) in rice, suggesting this miRNA plays a role in drought stress. There is a complex interplay between transcriptional and posttranscriptional regulation of drought response in Arabidopsis, but this has not been extensively characterized in tobacco.

The recent draft genome sequence of *Nicotiana benthamiana* [3] has provided a framework for the identification and functional characterization of genes and genetic networks for tobacco crop improvement and basic research. The availability of next-generation sequencing technologies, such as Digital Gene Expression (DGE) [24] and small RNA sequencing [25,26], together with the genome sequence, offer the opportunity to understand patterns of transcription in the context of plant responses to environmental stresses. Although several studies have documented individual genes that responded under drought-stress treatment of tobacco [27,28,29,30], a global study of transcription patterns of single or diverse organs using a single platform is lacking.

The root is generally the first organ in terrestrial plants that comes into contact with the drought stress. In this study, we used DGE [24,31], small RNA sequencing [32], and computational approaches to develop and map the transcriptional and posttranscriptional gene regulatory network that operates in response to drought stress in tobacco root tissue. This network analysis serves as a reference for future studies on tobacco response to various stresses, such as to drought, cold and heavy metals.

## 2. Results

### 2.1. Physiological Analysis of PRO, SOD, and MDA under Drought Conditions

**Figure 1:**
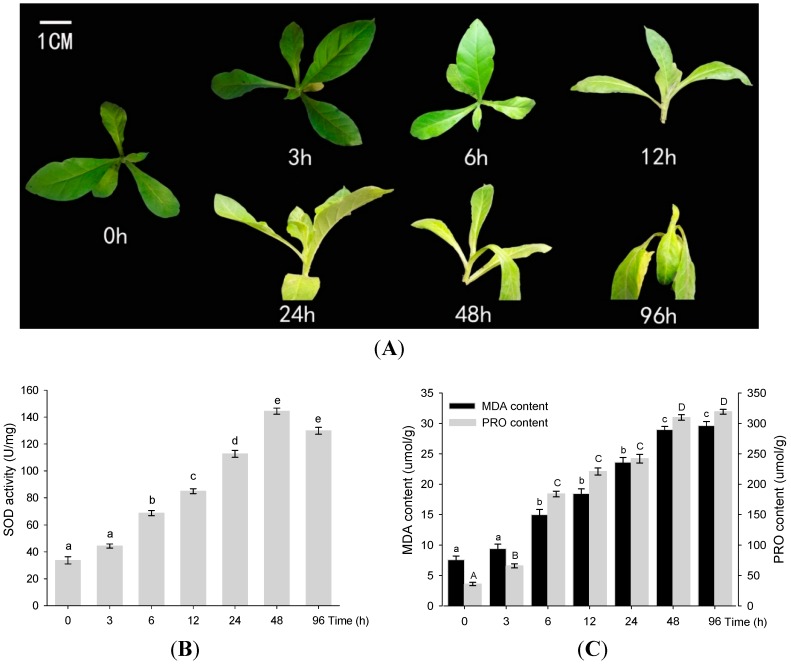
The growth status of tobacco under drought stress and corresponding physiological analyses. (**A**) The growth status of control plant (0 h) and drought-treatment plants at six time-points (3, 6, 12, 24, 48, and 96 h) with 20% PEG6000. The graphs (**B**) and (**C**) depicted the average change of superoxide dismutase (SOD) activities and contents of proline (PRO) and malondialdehyde (MDA), respectively, in the actual value ± standard error of the mean from control (time 0) across time for each treatment. Different lower or upper cases above standard error denoted the significantly difference of every two time –points (one-way ANOVA and Fisher’s *LSD*, *p* < 0.01, *n* = 3), whereas the same letter indicated no highly significant difference (*p* < 0.01). The gray bars in graph C represent the contents of PRO (µmol/g) and the black bars, the contents of MDA (µmol/g) at time 0 and six drought-treatment time-points. Time 0 represented the level obtained from the normal seedlings with six leaves.

To determine the optimal time to analysis gene expression for drought stress, uniform seedlings of tobacco with six leaves were challenged with 20% PEG6000 to simulate drought and then sampled at six durations of stress (3, 6, 12, 24, 48, and 96 h) (see “*Experimental Section*” for details). As shown in Figure 1A, the plants grew regularly under normal condition but leaves gradually turned yellow under longer duration of stress. After 48 h, plants were seriously damaged, and after 96 h they were wilted and dying. Superoxide dismutase (SOD) activities increased significantly (*p* < 0.01), and reached their peak after 48 h at about four times the control level (Figure 1B). This increase was paralleled by increased content of proline (PRO) and malondialdehyde (MDA) (Figure 1C), indicating that these three components in tobacco roots may act synergistically under drought stress. The general trend of the activity of SOD was different from that of PRO and MDA contents in that the former reached its peak at 48 h and then leveled off. PRO and MDA continued to ascend slowly and reached their peak after 96 h. These results suggest that SOD activity, PRO and MDA content all significantly increased at 6 and 48 h relative to 3 and 24 h, and the optimal times for drought stress assays were 6 and 48 h. Accordingly, our experiments of tobacco roots at 0 h (control) and two drought-treatment times were designated as NCK, N6H, and N48H.

### 2.2. Generation and Quality Assessment of the DGE Dataset

To document gene expression profiles of tobacco roots responding to drought stress, three DGE libraries (NCK, N6H, and N48H) were sequenced and high-quality sequences were obtained. The total sequence reads of all libraries ranged from 3.33 M (million) to 3.39 M (Supplementary Figure S1A and Table S1) with an average of 3.37 M. The N48H library had the highest number of distinct tags (242,065), followed by the NCK (229,344) and N6H (221,248) libraries (Figure S1B). Copy number of most of the distinct tags (over 67%) ranged from 2 to 5 (2 ≤ frequency ≤ 5). However, a small number of distinct tags (less than 2.2%) with frequency higher than 100 covered over 43% of all the clean tags in all three libraries (Supplementary Figure S1B).

When total tag number in NCK reached 1 million, the number of identified genes started to level off, and stabilized when the number of tags reached 3 million (Supplementary Figure S2A). The N6H and N48H data show a similar trend (Supplementary Figure S2A). This suggests additional distinct genes would not be identified after total clean tag number reached a certain value. Moreover, after sequencing depths reached 1 million tags, the number of distinct tags discovered dropped dramatically in all three libraries (Supplementary Figure S2B). In other words, increasing sequence depth resulted in a slow and stable accumulation of new distinct tags up to a point of saturation.

### 2.3. Mapping DGE Tags to the Tobacco Reference Genome Database

We used SOAP2 software [33] to map all distinct tags to the draft sequence of the *Nicotiana benthamiana* genome (v.0.4.4) [3], and the clean tags that matched perfectly or with only one mismatch were analyzed further. Mapping results showed that 73.40%, 72.81% and 70.80% of total clean tags mapped to the reference database (sense or anti-sense) for NCK, N6H and N48H, respectively, and 63.20%, 60.76% and 62.82% of reference genes were identified in NCK, N6H and N48H, respectively (Supplementary Table S1). In addition, around 30% of reads could not be mapped to the whole genome in three samples. In total, we detected 13,101 genes expressed in all three samples and 21,128 genes collectively (73.6% of the annotated transcriptome for tobacco) (Supplementary Figure S3).

### 2.4. Expression of Tobacco Root Genes under Drought Stress

**Figure 2:**
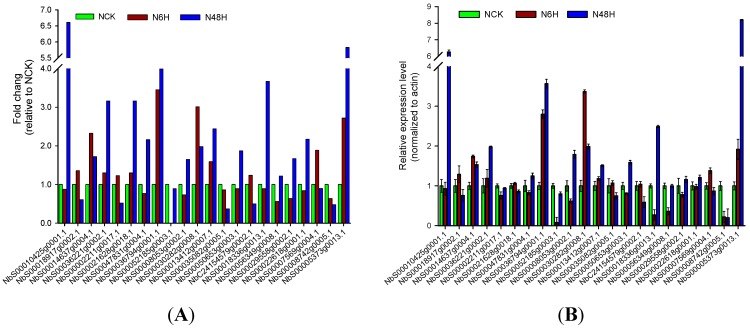
Expression of 22 genes in control and drought-treated roots of tobacco. (**A**) The levels of 22 genes detected by Solexa sequencing in three Digital Gene Expression (DGE) libraries (NCK, N6H and N48H); (**B**) The levels of these 22 genes detected by quantitative Real-Time PCR (qRT-PCR) in NCK, N6H and N48H (normalized to actin; *n* = 3). The green, purple and blue bars in graph B depicted the qRT-PCR relative expression level ± standard error of three replicates for each gene in NCK, N6H and N48H, respectively. NCK, N6H and N48H represented three samples of tobacco roots at 0 h (control, without drought-treatment) and two drought-treatment time-points (6 and 48 h), respectively.

We examined the dynamics of gene expression under drought stress using DGE data, and only 1887 out of 21,128 genes that were differentially expressed among NCK, N6H, and N48H, represented 8.9% of the root transcriptome (Supplementary Table S2). A sample of 22 genes with significant differences in gene expression was randomly selected for validation via qRT-PCR. For 10 of these genes, the expression dynamics of genes detected in the qRT-PCR experiments were indistinguishable from that based on three DGE data, and other 10 genes at least consistent with that obtained from these DGE data (Figure 2).

**Figure 3:**
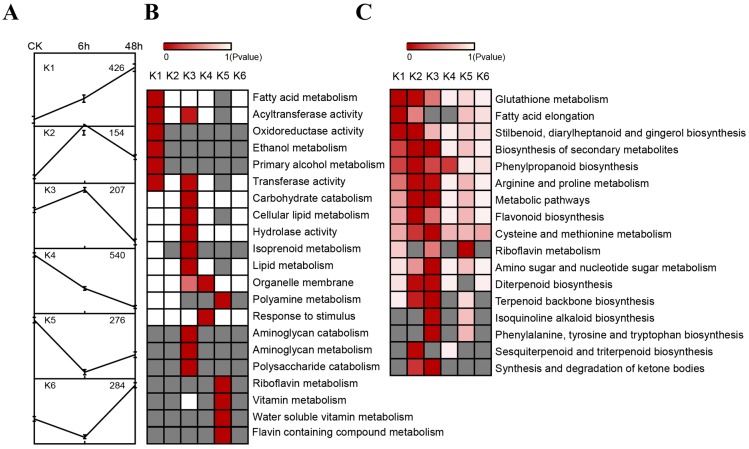
The dynamic profiles of all differentially expressed genes in tobacco roots on response to drought stress. (**A**) *K*-means clustering showing the expression profile of 1887 differentially expressed genes. Six clusters were identified in NCK, N6H and N48H from these genes above. These clusters are presented in **A**. Error bars show standard deviation; (**B**) Analysis of functional category enrichment among the six major clusters; (**C**) Analysis of pathways enrichment among the six major clusters.

We used Gene Ontology annotation to assign genes to functional categories and grouped genes by expression dynamics using the *K*-Means clustering algorithm [34]. We identified six clusters (Figure 3A,B and Supplementary Table S2). Genes that encode enzymes for fatty acid metabolism, transferase (transferring acyl groups), oxidoreductase, ethanol metabolism, primary alcohol metabolism and transferase were greatly enriched in cluster K1, including 426 genes, representing genes that were expressed at the highest levels in N48H. Genes with peak expression in N6H (clusters K3, 206 genes) were found to be required for transferase activity, carbohydrate catabolic process, cellular lipid metabolism, hydrolase activity, isoprenoid metabolic process, lipid metabolism, organelle membrane biosynthesis, aminoglycan metabolic process, and polysaccharide catabolic process, which suggests the root transcriptome undergoes drought stress first. Root gene expression was lowest in N48H (clusters K4, 539 genes) and was mostly restricted to genes involved in lipid metabolic processes. Genes encoding enzymes for polyamine metabolism, flavin containing compounds, polysaccharide, riboflavin, vitamin and water soluble vitamins showed a decline in expression after 6 h but reversed direction and their expressions were greatly enriched after 48 h (clusters K5, 275 genes).

In addition, biological pathways influenced by drought were evaluated by enrichment analysis of all differentially expressed genes. Significantly enriched metabolic pathways and signal transduction pathways were also identified. A total of 17 pathways were affected based on K1 thru K6 clusters (*p* < 0.05), and differentially expressed genes with pathway annotation were listed according to enrichment priority (Figures 3A,C and Supplementary Table S2). The expression level of genes in cluster K1 was lowest in the control but dramatically increased with time. These genes were associated with glutathione metabolism, fatty acid elongation, stilbenoid, diarylheptanoid and gingerol synthesis, synthesis of secondary metabolites, phenylpropanoid synthesis and arginine and proline metabolism (Figure 3C). Genes in the K2 cluster were mainly annotated as synthesis of secondary metabolites, flavonoid synthesis, stilbenoid, diarylheptanoid and gingerol synthesis, metabolic pathways, phenylpropanoid synthesis, glutathione metabolism, arginine and proline metabolism and sesquiterpenoid and triterpenoid synthesis (Figure 3C). The expression of genes in K3 cluster began to increase after 6 h and leveled off after 48 h. The K3 cluster with most of the enriched biological pathways affected by drought, included synthesis of secondary metabolites, terpenoid backbone synthesis, synthesis and degradation of ketone bodies, metabolic pathways, amino sugar and nucleotide sugar metabolism, phenylalanine, tyrosine and tryptophan synthesis, cysteine and methionine metabolism, diterpenoid synthesis, and isoquinoline alkaloid synthesis (Figure 3C). Although the genes in K4 and K5 showed a very high expression in the control, no significantly enriched pathways were found in K4, K5, and K6 clusters.

### 2.5. Resolving Transcription Factors (TFs) among Differentially Expressed Genes

**Figure 4:**
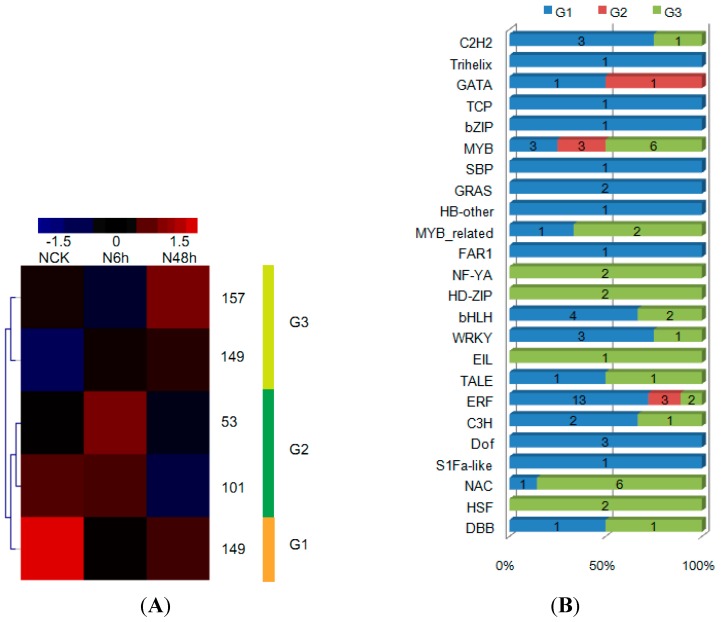
Dynamics of transcription factors in response to drought stress. (**A**) Dendrogram of the transcription factors. 609 expressed transcription factors from NCK, N6H and N48H were identified and clustered into three lineages (G1, G2 and G3) by *K*-means clustering (see “Materials and methods” for details); (**B**) Distribution of 82 differentially expressed transcription factor families among G1, G2 and G3.

A primary objective was to identify genes that encode TFs and elucidate the dynamics of accumulation of TFs under drought stress in our DGE data. To do this, we retrieved putative orthologs of tobacco genes based on information from the EnsemblCompara gene trees [35] at Sol Genomics network (solgenomics.net), PlantGDB (plantgdb.org), and NIBC (www.ncbi.nlm.nih.gov/). We then queried known plant TFs in the Plant Transcription Factor Database (v2.0, http://planttfdb.cbi.edu.cn/) and matched 609 tobacco TFs with sequence similarities to TFs of known plants. All TFs were detected in roots of tobacco seedlings in this study (Figure 4A and Supplementary Table S3A). Of these, 82 TFs, belonging to 24 families, were differentially expressed during stress (Figure 4B and Supplementary Table S3B). These TFs associated with functions in drought tolerance (MYB, NAC, and ERF) [7], development and meristem maintenance or identity (HD-ZIP, NF-YA, NAC, GRAS, and TCP) [36,37], defense/stress signaling pathways (HSP, WRKY, and bZIP) [38], hormone-mediated or stress-mediated signaling by auxin (AUX/IAA), brassinosteroids (BES), or ethylene and stress (AP2/ERF) [38]. Functional investigation of the roles of TFs was analyzed in detail in following sections.

The abundance of most of these TFs (53%) was highest in the control (G1), whereas 10% was highest after 6 h (G2). The reminder (37%) had peak expressions after 48 h (G3). We also identified family-specific expression trends (Figure 4B and Table S3B). Members of the C2H2 (three genes), MYB (3), bHLH (4), WRKY (3), ERF (13) and Dof (3) families of transcriptional regulators were highly expressed in the control. Several GATA (1), MYB (3) and ERF (3) TFs accumulated their highest levels after 3 h stress. Transcriptional regulators including MYB (6), NAC (6), MYB-related (2), NF-YA (2), HD-ZIP (2) and ERF (2) were preferentially expressed after 48 h of drought stress.

### 2.6. Identifying Candidate Drought Responsive Genes (DRGs) in Tobacco Roots

The 64 DRGs amalgamated from various drought studies on *Arabidopsis*, rice, tobacco, and maize (Table S4), were selected for this analysis. Most of these were signaling component genes acting upstream of TFs and the transcription factor encoding genes in the regulatory network of gene expression in drought and cold stress responses [6,7]. We identified 276 candidate DRGs in tobacco with sequence similarity to known genes and *Nicotiana benthamiana* annotation [3] (Table S4). About 40% (110 out of 276 genes) were TFs including WRKY, NAC, ERF, and bZIP families.

In our study, we also investigated the roles of these candidate DRGs, and found 46 differentially expressed DRGs under drought stress (Table 1 and Supplementary Figure S4). Of these, 21 (46%) were TFs which belonged to NAC (6), MYB (4), ERF (10), and bZIP (1) families. Other DRGs, such as GRF6, ABF1, APX2, SIPK, and ZPT2, have different expression patterns in response to drought stress.

**Table 1:** Possible drought responsive genes (DRGs) with different expression in tobacco roots.

Gene Name ^1^	Function	Homolog_Niben_ID	PlantGDB_or_unigenes_ID	Tranfactor_ID	Family	TPM-NCK	TPM-N6H	TPM-N48H
GRF6 (14-3-3 protein GF14 lambda)	Drought tolerance	NbS00060175g0001.1	-	-	-	161.74	80.67	123.31
		NbS00044217g0013.1	-	-	-	5.89	8.27	18.9
		NbS00020600g0007.1	-	-	-	22.98	13	33.3
		NbS00007737g0010.1	-	-	-	158.5	277.48	121.51
ABF1	Drought tolerance	NbS00021408g0025.1	-	-	-	0.88	18.03	0.6
ANAC055	Drought and salt tolerance	NbS00023955g0006.1	gnl|UG|Nta#S45443622	Nta005482	NAC	108.71	58.51	39
		NbS00008328g0013.1	gnl|UG|Nta#S45488905	Nta010847	NAC	35.06	60.28	73.51
ANAC072	Drought and salt tolerance	NbS00007567g0016.1	gnl|UG|Nta#S33559198	Nta013275	NAC	5.3	3.25	13.8
		NbC26152828g0003.1	gnl|UG|Nta#S45463754	Nta012057	NAC	23.57	48.76	99.01
		NbS00025931g0004.1	gnl|UG|Nta#S33566495	Nta005129	NAC	7.95	7.68	23.4
		NbS00028594g0003.1	gnl|UG|Nta#S45443622	Nta005482	NAC	19.15	21.87	56.7
APX2	Drought resistance	NbS00018810g0008.1	-	-	-	0.88	2.96	6.6
bZIP1	Drought, salt and disease tolerance	NbS00007503g0111.1	gnl|UG|Nta#S33578426	Nta007642	bZIP	84.55	40.48	28.5
CBF4	Drought and freezing tolerance	NbS00018604g0003.1	gnl|UG|Nta#S45471037	Nta012107	ERF	10.9	0.01	0.01
CpMYB10	Desiccation and salinity tolerance	NbS00016700g0002.1	gnl|UG|Nta#S45484072	Nta012264	MYB	2.36	10.05	1.8
		NbS00007512g0018.1	-	-	-	11.49	10.34	3.3
DREB1B/CBF1	Increased tolerance to drought, cold and salinity	NbS00009125g0101.1	gnl|UG|Nta#S33537030	Nta005632	ERF	114.31	11.52	16.2
DREB2A	Drought resistance	NbS00032542g0006.1	gnl|UG|Nta#S33577665	Nta005987	ERF	160.56	14.78	31.5
		NbS00025865g0001.1	gnl|UG|Nta#S33537030	Nta005632	ERF	13.26	0.89	7.5
		NbS00002425g0103.1	gnl|UG|Nta#S33577665	Nta005987	ERF	5.3	0.01	0.6
DREB3	Drought tolerance	NbS00016353g0001.1	-	-	-	20.03	3.25	2.7
FAD3	Drought resistance	NbS00037943g0006.1	-	-	-	17.97	27.48	42
		NbS00033197g0010.1	-	-	-	16.2	9.46	29.7
GmERF3	Drought tolerance	NbS00020925g0011.1	gnl|UG|Nta#S33555957	Nta004926	ERF	27.1	27.78	12.6
HSFA2	Resistance to environmental stresses	NbS00009669g0213.1	-	-	-	0.01	2.07	17.1
MYB60	Drought tolerance	NbS00046172g0003.1	gnl|UG|Nta#S40644996	Nta005483	MYB	1.77	1.77	12.3
		NbS00009475g0003.1	-	-	-	5.89	3.25	0.6
		NbS00009284g0003.1	gnl|UG|Nta#S33578151	Nta004281	MYB	65.4	108.75	28.8
		NbS00007772g0004.1	gnl|UG|Nta#S33578094	Nta008536	MYB	1.77	0.89	10.2
NCED1	Drought and salinity resistance	NbS00014845g0012.1	-	-	-	51.26	87.17	32.7
NtERD10B	Improved drought- and low temperature stress tolerance	NbS00041294g0010.1	-	-	-	0.01	0.01	7.5
		NbS00029572g0007.1	-	-	-	0.01	0.01	13.2
		NbS00019308g0009.1	-	-	-	2.36	5.61	86.41
NtERD10C	Improved drought- and low-temperature stress tolerance	NbS00018964g0003.1	-	-	-	75.12	12.41	21.9
SRK2E/OST1/SnRK2.6	Response to dehydration stress	NbS00019609g0014.1	-	-	-	3.83	7.98	18
		NbS00018358g0015.1	-	-	-	2.36	2.96	9.9
PP2Ac-1	Drought resistance; maintain RWC and membrane stability	NbS00043074g0007.1	-	-	-	12.67	8.57	21.9
		NbS00020903g0004.1	-	-	-	81.02	52.01	110.41
RD22	Response to drought stress	NbS00017616g0005.1	-	-	-	3.54	9.16	22.8
RD29A and RD29B	low-temperature-responsive and desiccation-responsive	NbS00001669g0005.1	-	-	-	9.43	3.84	1.8
SIPK	osmotic stress/pathogen resistance	NbS00060107g0004.1	-	-	-	36.53	42.85	78.91
WIPK	osmotic stress/pathogen resistance	NbS00041241g0001.1	gnl|UG|Nta#S37452582	Nta004233	ERF	7.66	0.59	1.5
		NbS00028162g0009.1	gnl|UG|Nta#S37452581	Nta012156	ERF	40.07	6.8	9.9
		NbS00017618g0003.1	gnl|UG|Nta#S37452582	Nta004233	ERF	58.33	5.91	2.1
ZPT2-1 (renamed from EPF1)	Drought tolerance	NbS00017486g0002.1	-	-	-	15.32	4.43	4.5
		NbS00002494g0018.1	-	-	-	44.78	33.1	19.8

^1^ For a version of the table with references for each gene, see Table S4. Abbreviations: *Nicotiana benthamiana*, Niben; TPM, transcripts per million clean tags.

### 2.7. Small RNA Sequencing

To examine miRNAs drought stress-response, two small RNA libraries were constructed based on the result of the physiological index measurement, and another two libraries from leaves and stems of the control plant. Four libraries were then sequenced using the Illumina high-through put sequencing technology. The resulting raw sequence reads (more than fourteen million for each library, 15–35 nt) were processed computationally to remove the 3'adapter and this yielded a total of 29,088,506 genome-matching reads (>18 nt) from the four libraries (6,939,963, 7,514,006, 7,331,406 and 7,303,131 reads from leaf, stem, and root controls, and treated root libraries, respectively) (Supplementary Table S5). The outstanding sequences that could not be mapped onto the tobacco genome were probably derived from unsequenced *Nicotiana benthamiana* genomic regions, sequence differences between *Nicotiana* species, sequence errors or contamination, and thus were excluded from subsequent analyses. More than 80% of these genome-mapped small RNAs ranged 20–24 nt reads in length with 24 and 21 nt as the modes which were consistent with the size of Dicer-like (DCL) cleavage products and similar to previous studies in other plant species (Supplementary Figure S5).

The genome-matched small RNA sequences were clustered into several RNA classes such as known miRNAs, repeats, rRNA, tRNA, snRNA, snoRNA and others (Supplementary Table S5). Known tobacco miRNAs account for 21.0% of all sequence reads for the leaf library (highest percentage) and 14.1% for the stem library (lowest percentage), suggesting that mature miRNAs were highly enriched in our small RNA libraries. However, after analyzing the number of unique sequences, the proportion of small RNA sequences derived from known miRNAs represented only a very small fraction (0.12%–0.16%) of the total number (Supplementary Table S5). The highest fraction of unique sequences (>93%) was unclassified small RNA sequences, which probably include novel miRNA candidates and other classes of regulatory RNAs.

### 2.8. Known Tobacco miRNAs Expressed in Different Tissues and Drought Responsive miRNAs in Tobacco Roots

**Figure 5:**
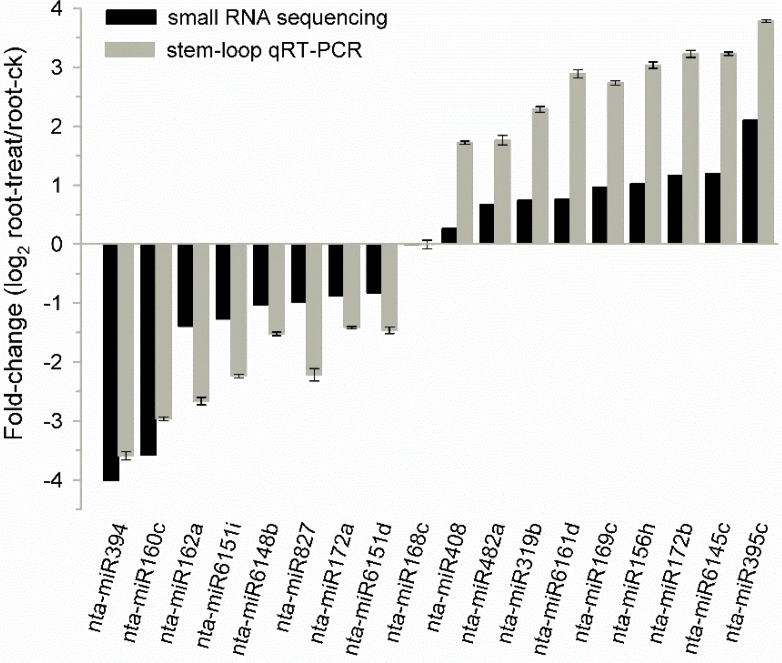
Expression of 18 miRNAs randomly selected in control and drought-treated roots of tobacco. The black bars represent the fold change (log2) in control library relative to drought-treated library detected by Solexa small RNA sequencing, while the gray bars represent the fold change (log2) in control roots relative to drought-treated roots detected by stem-loop qRT-PCR (normalized to 5S rRNA; *n* = 3).

Currently, miRBase (release 21 [39] contains 165 mature miRNAs sequences in tobacco. Of these, 122 were detected in our sequencing datasets (Supplementary Table S6) and all of the 18 miRNA families conserved in Arabidopsis and tobacco were included. Conserved miRNAs were far more abundant than non-conserved miRNAs in our libraries as reported previously [32,39,40]. MiR166 and miR168 were the most abundant miRNA families which accounted for about 57% and 16% respectively of the total sequence reads from the known miRNAs datasets. However, 44 experimentally identified tobacco miRNA families (33 miRNAs) were not detected in our dataset [25]. A sample of 18 expressed miRNAs was randomly selected for validation by stem-loop qRT-PCR. The expression trend of these miRNAs in control library relative to drought-treated library detected by Solexa small RNA sequencing were basically consistent with that detected by stem-loop qRT-PCR (Figure 5).

**Figure 6:**
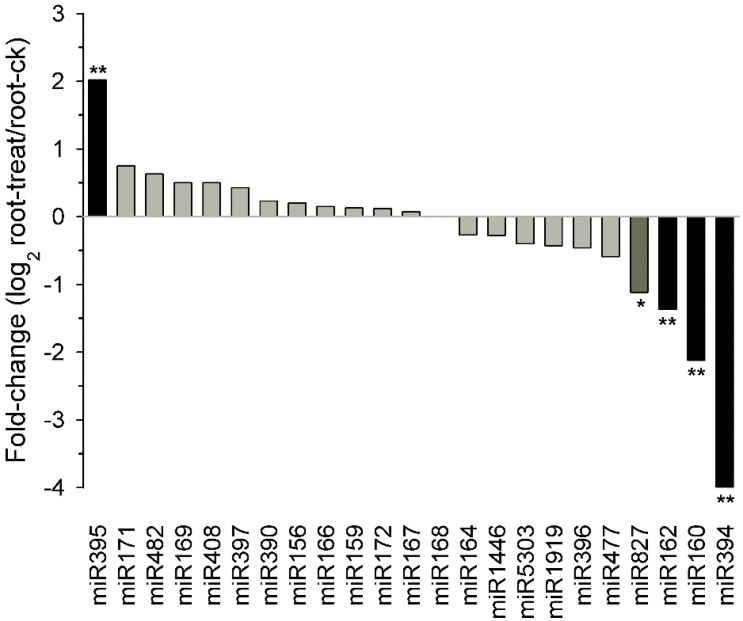
The expression profiles of the normalized sequence reads of the known miRNA families in the drought-treated library relative to the control library. * *p*-value < 0.01 is considered as significant; ** *p*-value < 0.001 is considered as extreme significant.

As reported [41], deep sequencing with good reproducibility is a powerful tool for analyzing genome-wide patterns of miRNA expression. The change in frequency of miRNAs between drought-treated and control libraries suggest that their expression was regulated in response to drought stress. As shown in Figure 6, comparison of the normalized sequence reads of the miRNAs between the two libraries indicated that 5 known tobacco miRNA families had relative changes (log_2_root-ck/root-treat) greater than other 18 miRNA families and thus might be differentially or extremely differentially expressed. However, miR159, miR169, miR402, and miR408 sequence reads displayed no meaningful changes between the two libraries (Figure 6) even though their expression had been reported to be affected by drought stress treatments in other plants [21,23,42,43].

### 2.9. Potential Targets of Drought Responsive miRNAs

miRNAs regulate gene expression at the posttranscriptional level by base paring with complementary sequences of mRNA thus inducing gene silencing or target degradation. Some miRNA families have been reported to respond to salt or drought stress in plants [42]. To understand the function of miRNAs’ response to drought in tobacco, we compiled a complete list of miRNA targets in tobacco identified by degradome sequencing [25]. Only 27 target GSS sequences, corresponding to 87 transcripts, could be mapped to the tobacco reference genome (Supplementary Table S7). Only targets of two drought-responsive miRNAs were obtained and listed in Table 2. Of these targets, miR160 and miR395 are highly conserved miRNA families and have been validated to target those mRNAs encoding ARF transcription factors and AST68 (Sulfate transporter 2.1) proteins, in *Arabidopsis* [44] and/or tomato [45], respectively. Previous studies showed that freeing *ARF17* or *ARF16* from miR160 regulation by transgenic *Arabidopsis* expressing miRNA-resistant targets resulted in dramatic morphological changes and alters basal levels of auxin-induced transcripts [46,47]. The expression of the validated targets of drought-responsive miRNAs was investigated by DGE data (Table 2). We found that there was a negative correlation for miR395 in the expression patterns of the miRNAs and their targets. This is consistent with miRNA function in guiding the cleavage of target mRNAs.

**Table 2:** Drought responsive miRNA families and their targets in tobacco.

miRNA_family	Regulated	Target_GSS_ID	Mapped_Niben_ID	NCK_TPM	N6H_TPM	N48H_TPM	Description
miR160	down	ET792241	NbS00018737g0003.1	0.59	1.18	1.20	ARF Transcription factor
miR160	down	ET792303	NbS00005871g0012.1	0.01	0.01	0.60	ARF Transcription factor
miR160	down	FH226342	NbS00059497g0003.1	4.71	3.84	4.20	ARF Transcription factor
miR160	down	FH226342	NbS00026905g0003.1	6.78	3.84	4.50	ARF Transcription factor
miR160	down	FH226421	NbS00028965g0003.1	3.54	2.66	1.20	ARF Transcription factor
miR160	down	FH226421	NbS00036438g0004.1	0.01	0.59	0.01	ARF Transcription factor
miR395	up	ET937275	NbS00014405g0001.1	2.36	1.48	0.90	unknown protein
miR395	up	ET937275	NbS00009197g0109.1	12.37	3.25	2.40	unknown
miR395	up	ET937275	NbS00006001g0005.1	11.78	17.43	2.10	unknown protein

## 3. Discussion

We first performed DGE analysis to determine genome-wide patterns of gene expression of tobacco roots under drought stress. Given the nature of the DGE system, we pooled biological replicates for each group to make representative samples for deep sequencing analysis based on physiological analysis of PRO, SOD, and MDA. We detected expression of 21,128 genes, which accounted for 73.6% of predicted genes in the tobacco genome. Approximately 30% of the clean tags could not be mapped to any genes in all three samples. These non-mapped tags most likely represent regions where the tobacco reference sequence is incomplete [3], or there are allelic sequence differences between the reference genome and the cultivar used in this study. Alternatively, differential mRNA processing events exist for most tobacco genes, such as alternative splicing [34]. Another reason may be that RNA-seq data for tobacco genome annotation should represent all major tissue types, developmental stages and responses to abiotic and biotic stresses [48]. If the annotated transcripts contained SNP, RNA editing and InDel which are located in CATG sites by chance, and, therefore, the reads could not be mapped to the reference genome sequence.

Through the use of DGE, this global analysis of gene expression provided a comprehensive dataset responding to drought stress in roots of tobacco seedlings. We identified six clusters for all differentially expressed genes and coarsely assigned them to 21 functional categories (*p* < 0.05) (Figures 3A,B and Supplementary Table S2). Interestingly, there were no other overlapping GO functional enrichments between clusters except transferase, transferring acyl groups, indicating that these genes of different clusters were predicted to be involved in many plant biological processes, including defense [6].

Pathway enrichment analysis revealed 17 pathways were significantly affected by drought stress (Figure 3A,C and Supplementary Table S2). These pathways were enriched in K1/K2/K3 clusters. Glutathione metabolism and biosynthesis of secondary metabolites pathways were the most affected in K1 and K2/K3 clusters, respectively. The former finding implied that Glutathione (GSH) was an abundant and ubiquitous antioxidant with proposed roles in the maintenance of tissue antioxidant defenses and in the regulation of redox-sensitive signal transduction. The size of glutathione pool and its redox status were tightly correlated with the tolerance of plants to drought stress [49,50]. Genes that function in fatty acid (FA) biosynthesis, elongation and lipid beta-oxidation were also enriched in FA elongation of K1, suggesting an elaboration of plastid machinery under drought stress. A number of genes annotated as biosynthesis-related (e.g., NAD(P)-binding Rossmann-fold superfamily protein (NbS00002389g0014.1), Glucose/ribitol dehydrogenase (NbS00007017g0016.1) and ADH-like UDP-glucose dehydrogenase (NbS00019080g0006.1)) were enriched in biosynthesis of secondary metabolite pathway in cluster K2/K3, which was previously described as plant defense-related pathway [51]. We also noted two differentially expressed genes (Aquaporin (NbS00007456g0005.1) and Glutathione transferase, (NbS00049661g0007.1)), whose homologs in other plants play an important role in drought tolerance [52,53,54] and oxidative stress [55], respectively.

Previous studies have uncovered gene expression, transcriptional regulation, and signal transduction in plant responses to drought [6]. In the signal transduction network that leads from the perception of stress signals to the expression of stress-responsive genes, transcription factors (TFs) play an essential role [7]. In our study, TFs detected in DGE analysis included 82 members of 24 TF families with differential expression, of which 21 were DRGs, indicating that these TFs probably played important roles in response to drought.

In addition, some miRNAs have been shown to be stress regulated at the posttranscriptional level by repressing mRNA expression and could be involved in cell responses to various abiotic stresses such as salinity and drought [42]. Some miRNA target genes are stress-responsive TFs or functional genes [42], indicating that miRNA-dependent posttranscriptional regulation may play a role in plant stress response. In this study, we used high-throughput sequencing to confirm expression of the known miRNAs and their changes upon drought treatment. We detected the expression of 122 known miRNAs (39 families) in tobacco roots, stems and leaves. Upon drought treatment, five conserved miRNA families showed differential regulation (Figure 6). One of these drought-responsive conserved miRNA families, miR395, has been shown to be stress responsive in other plant species [42], suggesting that these miRNAs might also play a positive regulatory role in drought-tolerance in tobacco roots. Additionally, most targets of the new miRNAs identified by degradome sequencing [25] were mapped to the reference tobacco genome and 87 potential target genes were found. Ten targets responsive to drought stress suggest the importance of these miRNAs in stress responses in tobacco (Supplementary Table S7). However, expression of some miRNA families (miR159, miR169, miR402 and miR408) had no meaningful changes between libraries thus indicating that different stress conditions might evoke diverse plant responses and physiological adaption.

**Figure 7:**
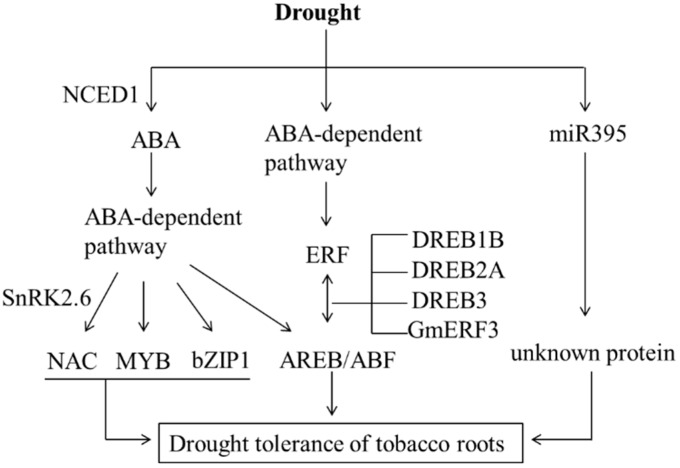
A probable model for regulatory network being involved in drought responses in tobacco roots. The integrated gene regulatory network has been proposed on basis of previous regulatory network model of Arabidopsis in the drought and cold stress responses. Abbreviations: ABA, abscisic acid; NCED, 9-*cis*-epoxycarotenoid dioxygenase gene; SnRK2.6, SNF1-related protein kinase2.6/OST1 kinase; NAC, NAM, ATAF, and CUC transcription factor; bZIP, basic-domain leucine-zipper; ABF, ABRE-binding factor; AREB, ABA-responsive-element-binding protein; ERF, ethylene-responsive-element-binding factor; DRE, dehydration-responsive element; DREB, DRE-binding factor; DREB1B, DREB2A, DREB3 and GmERF3 are the members of ERF family.

With the availability of regulatory networks of gene expression in drought and cold stress responses [6], an integrated gene regulatory network has been proposed for the molecular mechanisms of the response of tobacco roots to drought stress using differentially expressed DRGs, the changed expression profiles of miRNAs and subsequent target transcripts as a basis [56] (Figure 7). Two pathways (ABA-dependent and ABA-independent) can shed light on cell mechanisms involved in stress signaling and/or adaptation at transcriptional regulation. In the ABA-dependent pathway, NCED1 was involved in rapid emergency response to drought and Figure 7 shows transcription cascades involved in slow and adaptive processes in stress responses, such as those involving AREB/ABF, MYB, bZIP, NAC and SnRK2.6 protein kinases which are involved in ABA signaling [57,58]. In the ABA-independent pathway, unknown proteins were thought to function as an osmo-sensor and function upstream of the ERF system [59]. In addition, the responsive miR395 also showed a transitory expression model. Right side of Figure 7 shows the proposed regulation cascades in tobacco roots after initiation of drought stress. Our study provides valuable information for future studies of the molecular mechanisms underlying drought tolerance in tobacco roots and other plants.

## 4. Experimental Section

### 4.1. Plant Materials, Growth Conditions and Stress Treatments

A flue-cured tobacco (*Nicotiana tabacum* L.) cultivar named Honghua Dajinyuan, known for drought-tolerance and widely grown in Southwest China, was used in our study. The seed was sterilized and germinated in an incubator as reported earlier [60]. The germinated seed was then sown in spots with soil matrix and grown until the seedlings developed six leaves at 28/21 °C day/night temperatures (16 h day/ 8 h night) and relative humidity of 70%. To mimic drought, seedlings were treated by adding 20% PEG6000 to the matrix. Samples were taken 0, 3, 6, 12, 24, 48, and 96 h after treatment [60]. The control was not treated and is the time zero treatment. Roots were collected from all treatments. In addition, leaf and stem samples were collected from the control. Tissues were snap-frozen in liquid nitrogen, and then stored at −80 °C for physiological index measurements [61,62,63], RNA extraction, and quantitative real-time PCR (qRT-PCR) validation.

### 4.2. Physiological Analyses

Ultraviolet-visible transmittance spectrophotometers (UV-VIS) (Shimadzu, UV-2450, Kyoto, Japan) were used to measure three physiological indexes: superoxide dismutase (SOD) activity, and proline (PRO) and malondialdehyde (MDA) content of the root samples [61,62,63]. A physiological assay kit was used and tests were performed according to the manufacturer’s protocol (Nanjing Jiancheng Bioengineering Institute, Nanjing, China). All tests were replicated three times. Single factor analysis of variance was performed using SPSS16.0 (SPSS Inc., Chicago, IL, USA). Fisher’s Least Significance Difference (LSD) with multiple comparisons was used to separate treatments at (*p* < 0.01, *n* = 3) level of significance.

### 4.3. DGE, Small RNA Library Construction, and Solexa Sequencing

Total RNA was isolated from the frozen roots samples by using Trizol Reagent (Invitrogen, Carlsbad, CA, USA) according to manufacturer’s instructions. DGE library preparation was performed in parallel by using the Illumina gene expression sample preparation kit as described by [31,64]. Each tunnel will generate millions of raw reads with sequencing length of 35 bp. Raw data (tag sequences) were deposited in the GEO database (No. GES43058).

The samples of our small RNA libraries were used based on the result of physiological index measurement as follows: equal quantities (10 µg) of total RNA isolated from tobacco roots treated with two time points (6 and 48 h) were mixed together to construct the drought-treated small RNA library (Root-treat), and total RNA prepared from the control roots sample was used to construct the control small RNA library (Root-ck). In addition, we constructed two libraries from leaves and stems of the control plants. These libraries were constructed using the Small RNA Sample Prep Kit (Illumina, San Diego, CA, USA) as described by Tang *et al.* [25] and Yin *et al.* [32].

### 4.4. Analysis and Mapping of Digital Gene Expression Tags

Raw sequencing image data were transformed by base calling into sequences. Raw data reads were stored in FASTA format, and analysis was conducted as described by Shen *et al.* [31,64]. Prior to mapping to the reference database, all sequences were filtered to trim the 3' adaptor sequence, filter empty tags (reads with only 3' adaptor sequences but no tags) and low-quality tags containing Ns, and remove tags which are too long or too short. Because of the lack of EST sequence annotation of *Nicotiana tabacum* L., we chose the genome of its close relative *Nicotiana benthamiana* (v.0.4.4) [3] as reference, which is a virtual library containing all possible CATG + 17 base-length sequences and present high degree of sequence similarity (>90%) with *Nicotiana tabacum* L. (Supplementary Figure S6). All clean tags were mapped to the reference sequences and a mismatch of only 1 bp was considered. Clean tags that were mapped to the tobacco genome reference sequences from multiple genes were filtered. The remaining clean tags were designated unambiguous clean tags. The expression level of each gene was estimated by the frequency of clean tags and then normalized to TPM (transcripts per million clean tags) [65], which is a standard method and extensively used in DGE analysis [66]. The expression level of each gene was measured by the normalized number of matched clean tags. KOG functional classification, Gene Ontology (GO), pathway annotation and enrichment analyses were based on the Clusters of Orthologous Groups of proteins (COGs) (http://www.ncbi.nlm.nih.gov/COG) [67], Gene Ontology Database (http://www.geneontology.org/) [68] and Kyoto Encyclopedia of Genes and Genomes (KEGG) pathway (http://www.genome.jp/kegg/) [69], respectively. When we investigated pathways in which different genes were involved and enriched, *q*-value was used to aid identification according to the previous description [70].

### 4.5. Identification of Differentially Expressed Genes and Cluster Analysis

For differential expression analysis between control and treatment samples, the probability that one gene G is equally expressed in two samples was expressed by *p* values [71]. Thresholds of *p* values for multiple tests were determined by False Discovery Rates (FDR) [72]. In addition, we applied the R package [73] to identify differentially expressed genes with the random sampling model based on the read count for each gene under drought stress. We used “FDR ≤ 0.001 and the absolute value of log2-fold change ≥ 1” [74] as the threshold for judging the significance of different gene expressions. More stringent criteria with a smaller FDR and higher fold-change value were used to identify differentially expressed genes. GO functional enrichment analysis of differentially expressed genes was carried out using Blast2GO (version 2.3.5, https://www.blast2go.com/). KEGG pathway analyses of differentially expressed genes were performed using Cytoscape software (version 3.1.1, http://www.softpedia.com/get/Science-CAD/Cytoscape.shtml) with the ClueGO plug (http://www.ici.upmc.fr/cluego/cluegoDownload.shtml) [75]. GO annotations were performed using AgriGO (version 1.2; http://bioinfo.cau.edu.cn/agriGO/). *K*-means clustering was previously described by Li *et al.* [34].

### 4.6. Analysis of Small RNA Sequencing Data and Identification of Drought Responsive miRNAs

The overall procedure for analyzing Solexa small RNA libraries was performed as previously described [25,26]. The Illumina Gerald pipeline was used to process and extract the first 36 bases of each read. Adaptor sequences were identified and trimmed from each read using a customized Perl script. Reads in which the adaptor could not be identified were discarded. We used the *Nicotiana benthamiana* genome (v.0.4.4) database (ftp://ftp.solgenomics.net/genomes/Nicotiana_benthamiana) [3], and Sanger Rfam data (ftp://ftp.sanger.ac.uk/pub/databases/Rfam/) [76] to identify sequences originating from protein-coding genes, repeats, rRNA, tRNA, snRNA, and snoRNA. SOAP2 software [33] was used to align the trimmed reads to the high-confidence set of 165 pre-miRNAs from miRBase (version 21; http://www.mirbase.org/) [25]. For each library, we counted the number of trimmed reads within the 18–25nt ranges that were mapping to each pre-miRNA and normalized by the total number of 19–24 nt trimmed reads in the library. Trimmed reads that were < 18 or ≥ 25 nt were not considered in this analysis. The relative change of individual miRNA families of detected reads for treatments and *p*-values of the *t*-test were calculated; differentially expressed miRNA families were those with *p*-values <0.01.

### 4.7. Quantitative Real-Time PCR (qRT-PCR) Analysis

In order to verify the DGE sequencing results, 22 differentially expressed genes were randomly selected and qRT-PCR performed (Supplementary Table S8A). Specific primers for an internal gene, *actin* (GenBank no. X63603, Forward: 5'-CGCGAAAAGATGACTCAAATC-3', Reverse: 5'-AGATCCTTTCTGATATCCACG-3'), were used for the normalization of reactions [77]. qRT-PCRs were performed using SYBR Premix Ex Taq protocol (TaKaRa, Shiga, Japan) on an Applied Biosystems 7500 Real-Time PCR System (Applied Biosystems, Foster City, CA, USA). RNA samples were the same as those for the DGE experiments from biological replicates, and triplicate experiments were performed using independent plant materials, from which results were calculated using the 2**^−^**^ΔΔ*C*t^ method [78].

Expression trends of 18 drought responsive mature miRNAs were assayed by stem-loop reverse transcription-PCR (RT–PCR). 200 ng of total RNA was used for the initiation of the reverse transcription reaction. The stem–loop reverse transcription primers were designed following the method described by Chen *et al.* [79] and Varkonyi-Gasic *et al.* [80]. The reverse transcription product was amplified using a miRNA-specific forward primer and a universal reverse primer. The stem–loop reverse transcription reactions were performed using One Step PrimeScript^®^ miRNA cDNA Synthesis Kit (TaKaRa, Japan), and PCR primers were then added to perform the PCR on the Applied Biosystems 7500 Real-Time PCR System (Applied Biosystems, Foster City, CA, USA). One of the uniformly expressed 5S rRNAs was used as the internal control for stem-loop qRT-PCR [81]. Supplementary Table S8B shows the sequences of stem-loop RT primers and miRNA-specific PCR primers.

## 5. Conclusions

In summary, this study provided a comprehensive analysis of drought-responsive genes and microRNAs expression profiles of tobacco roots by combined DGE, small RNA sequencing, and computational approaches, and revealed 54 differentially expressed DRGs included 21 TFs, which belonged to 4 TF families such as NAC (6), MYB (4), ERF (10), and bZIP (1). Additionally, we confirmed expression of 39 known miRNA families (122 members) and five conserved miRNA families, which showed differential regulation under drought stress. Targets of miRNAs were further surveyed based on a recently published study, of which ten targets were DRGs. Finally, we developed and mapped the transcriptional and posttranscriptional gene regulatory network that operated in response to drought stress in tobacco root tissue. This network analysis provided new clues for future studies on tobacco response to various stresses, such as to drought, cold and heavy metals.

## Supplementary Materials

Supplementary materials can be found at http://www.mdpi.com/1422-0067/16/03/5714/s1.
