# Supplementary material for: Genome-Wide Identification and Analysis of Drought-Responsive Genes and MicroRNAs in Tobacco
Source: Int J Mol Sci. 2013 Mar 12;16(3):5714–40. doi: 10.3390/ijms16035714 (PMC4394501; doi:10.3390/ijms16035714)
Supplement: Supplementary file 1 [file ijms-16-05714-s001.zip › ijms-72460-Supplementary Information-Figures.pdf]

Supplementary Information

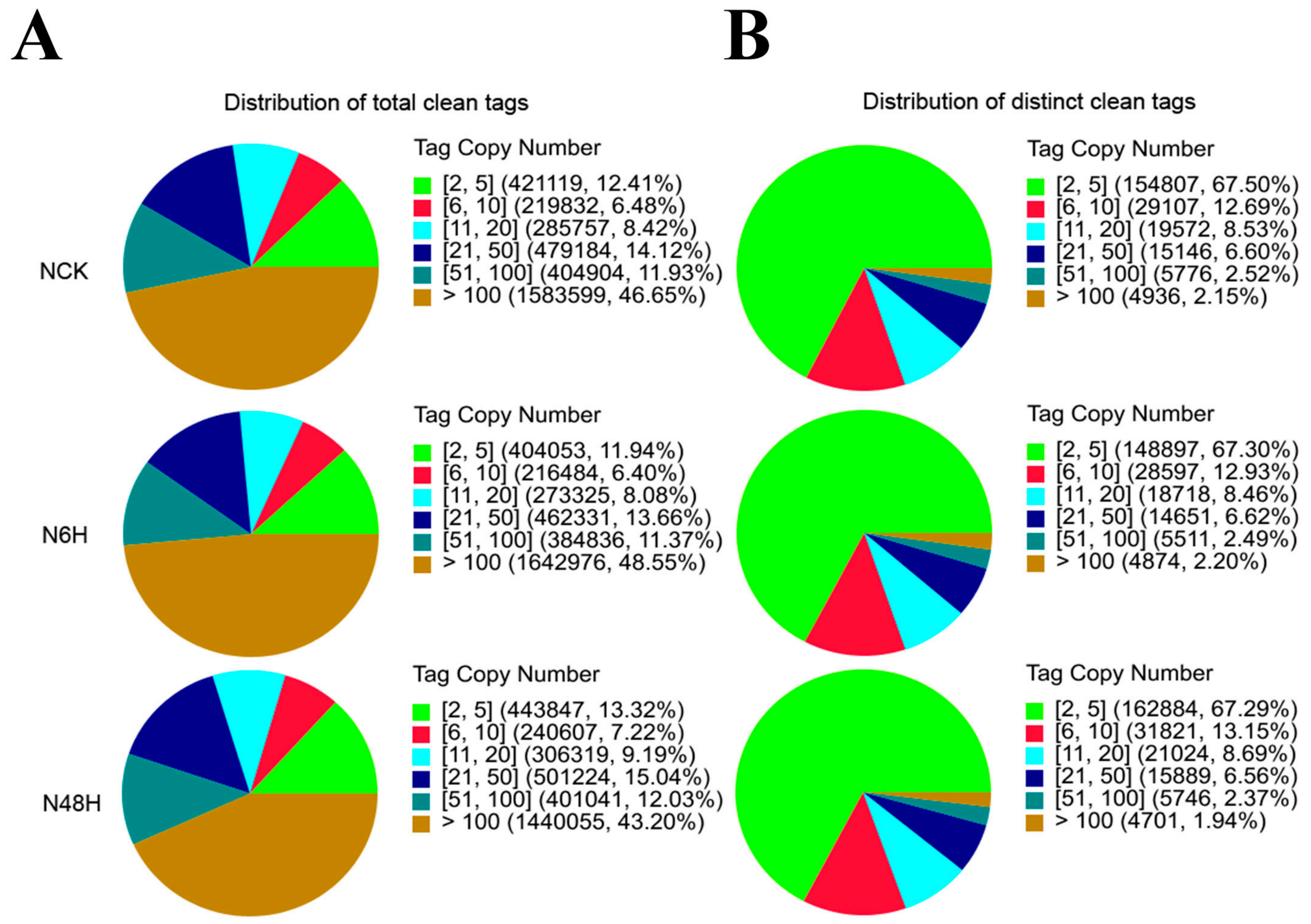

Figure S1. Distribution of total (**A**) and distinct clean tags (**B**) over different tag-abundance categories in three DGE libraries (NCK, N6H and N48H).

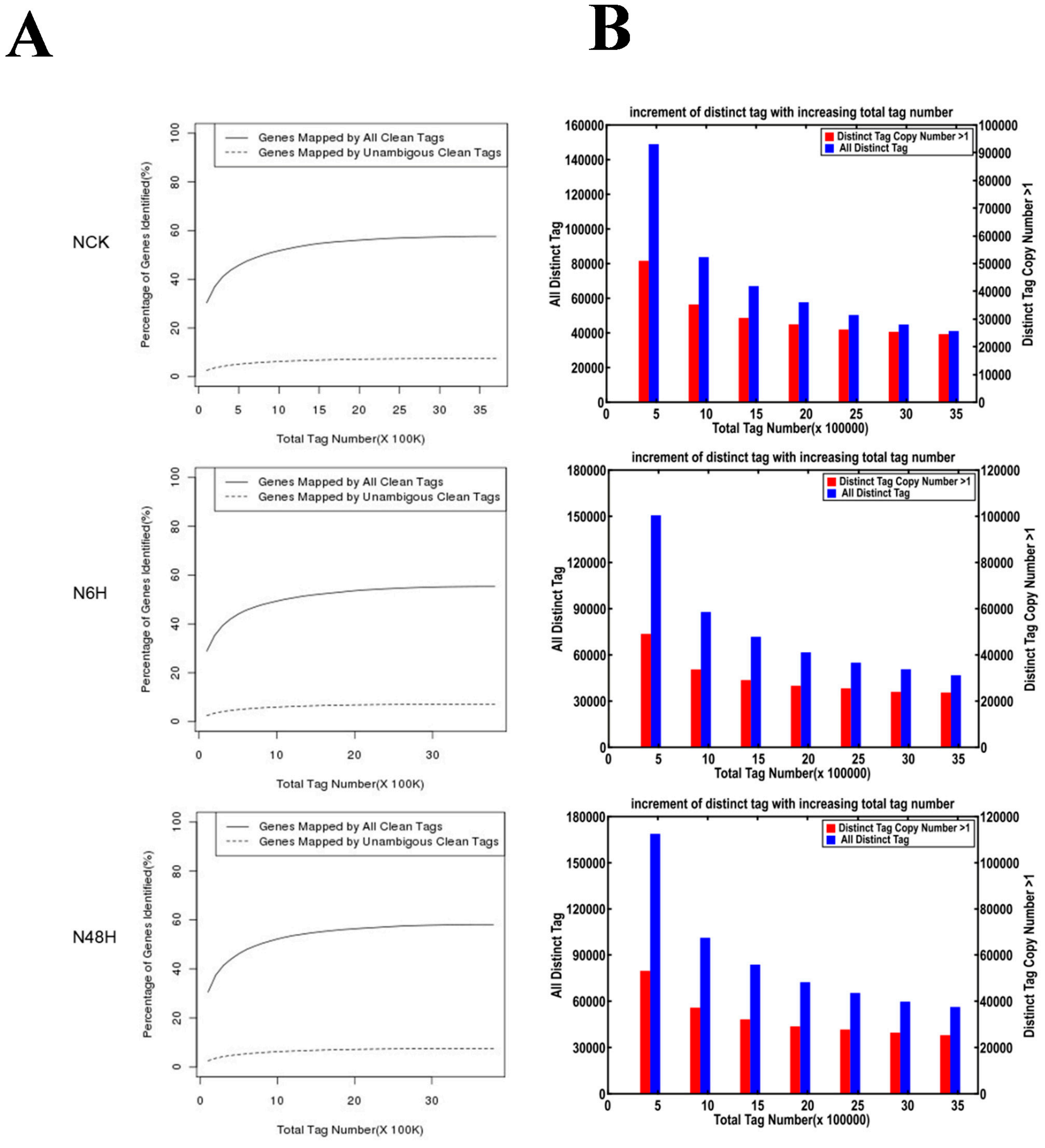

Figure S2. Saturation of three DGE libraries (NCK, N6H and N48H).

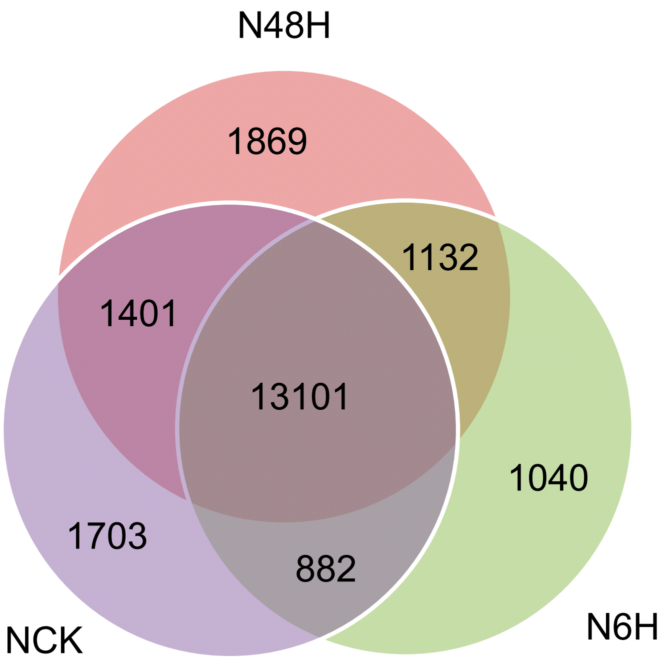

Figure S3. Venn diagram of all expressed genes from three DGE libraries (NCK, N6H and N48H).

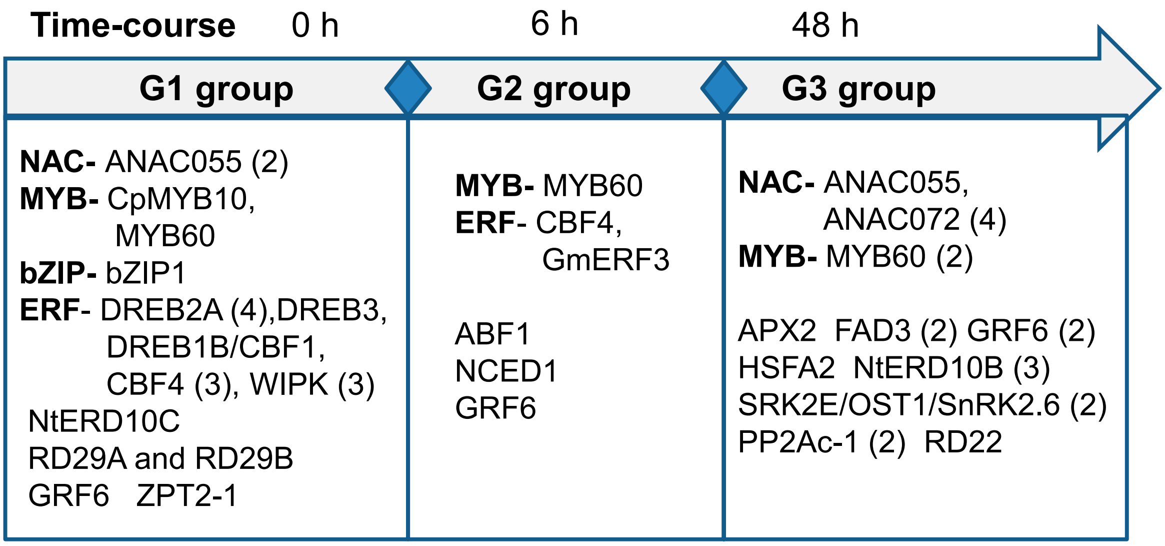

Figure S4. Differentially expressed drought responsive genes (DRGs) showing expression gradients along time-course. The number in bracket represents the number of genes which have the similar function as adjacent gene experimentally validated in other plants. Abbreviations: ABF, ABRE-binding factor; AREB, ABA-responsive-element-binding protein; ERF, ethylene-responsive-element-binding factor; DRE, dehydration-responsive element; DREB, DRE-binding factor. CBF4, DREB1B, DREB2A, DREB3, *GmERF3* and WIPK are the members of ERF family; APX, L-ascorbate peroxidase; bZIP, basic-domain leucine-zipper; GRF, growth-regulating factor; FAD, fatty acid desaturation; HSFA, class A of heat shock transcription factor; *NCED*, 9-cis-epoxycarotenoid dioxygenase gene; SnRK2.6, SNF1-related protein kinase2.6/OST1 kinase; NAC, NAM, ATAF, and CUC transcription factorPP2Ac-1, catalytic subunit (c) of protein phosphatase 2A; ZPT, Renamed from EPF, Cys2/His2-type zinc finger protein.

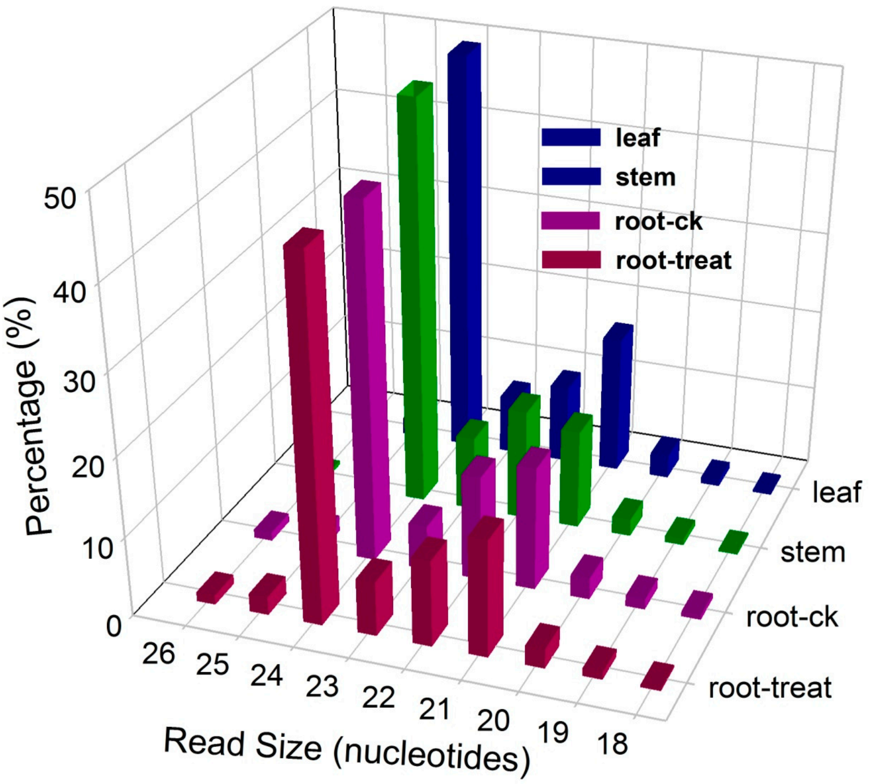

Figure S5. Length distribution of small RNAs from different tissues (roots, stems and leaves) under the same normal conditions and drought-treated roots.

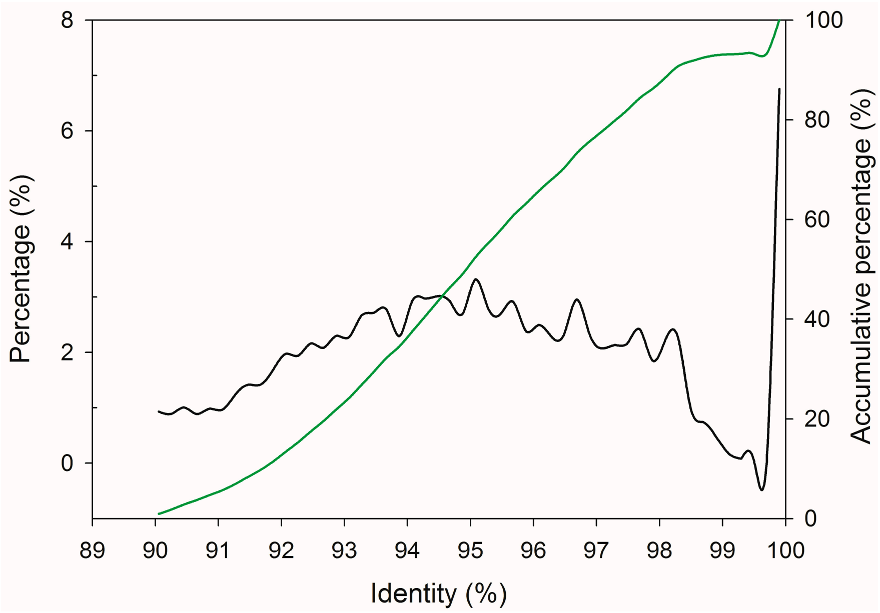

Figure S6. The identity of ESTs sequence from *Nicotiana tabacum* L. and the genome database from *Nicotiana benthamiana.* The black curve line is the percentage of ESTs with identity >90%, while the green curve line is accumulative percentage of ESTs with identity >90%.
